# Continuous Vital Signs Monitoring with a Wireless and Wearable Earsensor in Surgical Patients: A Clinical Validation Study

**DOI:** 10.3390/s26041201

**Published:** 2026-02-12

**Authors:** Patrick van den Berge, Kim van Loon, Lianne Zevenbergen, Pascalle A. van den Heuvel, Martine J. M. Breteler

**Affiliations:** 1 Department of Anesthesiology, University Medical Center Utrecht, Utrecht University, 3508 TC Utrecht, The Netherlands; 2 FastFocus B.V., 3481 LT Harmelen, The Netherlands; 3 Department of Technical Medicine, University of Twente, 7500 AE Enschede, The Netherlands; 4 Department of Internal Medicine, University Medical Center Utrecht, Utrecht University, 3508 TC Utrecht, The Netherlands

**Keywords:** remote patient monitoring, wearable device, photoplethysmography, vital signs, clinical deterioration

## Abstract

(1) Background: Evidence on the clinical accuracy of wireless photoplethysmography (PPG)-based vital sign monitoring is limited. This study evaluated the accuracy, technical performance, and patient comfort of a novel PPG-based earsensor for measuring oxygen saturation (SpO_2_), pulse rate (PR), and respiratory rate (RR) in postoperative patients. (2) Methods: In this observational method comparison study, SpO_2_, PR, and RR were simultaneously recorded using the earsensor and compared with continuous monitoring in patients admitted overnight to the post-anesthesia care unit. Outcome measures were bias, 95% limits of agreement (LoA), and average root mean square (ARMS). Technical performance was evaluated by data loss and data gap duration. Patient comfort was assessed using a questionnaire. (3) Results: Twenty-one patients contributed to 264 h of data. Bias was 1.7% for SpO_2_ (ARMS 2.4%; LoA −1.8% to 5.1%), 1.2 bpm for PR (ARMS 3.9 bpm; LoA –6.1 to 8.4 bpm), and 0.3 brpm for RR (ARMS 4.4 brpm; LoA –8.4 to 8.9 brpm). Overall, data loss was 42% for SpO_2_, 33% for RR, and 29% for PR; most data gaps were under 30 min. Patient-reported comfort was high (77%). (4) Conclusions: The earsensor accurately measured SpO_2_ and PR. RR accuracy was outside the predefined criteria. Despite substantial data loss, patient comfort was high, supporting the potential of PPG-based sensors for unobtrusive vital sign trend monitoring in low-acuity settings.

## 1. Introduction

Although certain patient complications can occur suddenly, most cases of patient deterioration develop gradually over time. Adverse events and physiological instability are often preceded by abnormal vital sign deviations and other warning signs [1,2,3]. Close monitoring supports the detection of early changes in physiological patterns. However, in many hospital practices, nurses and physicians typically rely on intermittent spot checks performed once every 8 h shift. Compliance with early warning score (EWS) protocols is often suboptimal, with measurements being incomplete, delayed, or not executed at all [4,5,6]. Consequently, early signs of deterioration may remain unnoticed until the next scheduled observation [7,8], potentially resulting in preventable adverse events and increased healthcare costs [9,10].

Wearable wireless sensors for continuous vital signs monitoring offer a promising solution to healthcare professionals in detecting patient deterioration both in hospital wards and at home. These technologies provide access to vital sign trend data, facilitate earlier recognition of clinical changes, and support shared decision-making [11,12]. Over the past decade, many wearable sensors that entered the market use vital sign measurements derived from electrocardiography (ECG), typically placed on the chest. The COVID-19 pandemic has further accelerated the adoption of continuous monitoring, particularly the use of pulse oximetry to monitor early signs of hypoxemia.

Pulse oximeters rely on photoplethysmography (PPG) sensors placed on well-perfused parts of the body, such as the finger, toe, or earlobe, to optimize pulse amplitude and improve measurement accuracy. Traditional ‘wired’ pulse oximeters are highly susceptible to motion artifacts, particularly during patient movement, which limits their suitability for continuous monitoring in ambulatory or mobile patients [13]. Wireless PPG-based sensors have, therefore, become increasingly popular for continuous monitoring of multiple vital signs, including oxygen saturation (SpO_2_), respiratory rate (RR), and pulse rate (PR), and nowadays represent the most commonly used technology in wearable monitoring systems [14,15]. This development has fueled interest in wearable PPG sensors that are both robust to motion artifacts and comfortable enough for prolonged, unobtrusive use.

Despite a growing interest, evidence on the accuracy of medical-grade wireless sensors capable of continuous SpO_2_ monitoring remains limited, even though it is known that up to 80% of desaturation episodes are reportedly missed with standard intermittent monitoring on general wards [16]. Furthermore, ECG-based measurements of HR and RR are generally considered more precise than those derived from PPG signals [17,18]. Only a small number of studies have evaluated wireless, wearable PPG-based sensors for measuring vital signs in clinical settings. One study assessed a device during heart catheterization procedures, where patient movement was minimal, thereby limiting generalizability to routine clinical use [19]. Another study evaluated a device over a brief 30 min monitoring period in postoperative patients [18]. Other studies have assessed consumer-grade pulse oximeters; however, these devices are intended primarily for spot checks rather than continuous measurements [20,21].

Currently, there are no validated wearable and wireless pulse oximetry sensors capable of continuously monitoring vital signs from alternative body sites beyond the wrist, finger, or upper arm. An accurate, comfortable, vital signs earsensor with high patient acceptability could fill this void. However, before proceeding to larger multicenter trials studying patient outcomes, it is crucial to validate vital sign measurement performance in clinical practice [22]. Therefore, the objective of this study is to evaluate the accuracy of the PPG-based oxygen saturation (SpO_2_), pulse rate (PR), and respiratory rate (RR) measurements obtained using a medically certified PPG-based earsensor (Vital Signs Monitoring System, FastFocus B.V., Harmelen, The Netherlands), and to assess patient comfort and technical performance in postoperative patients.

## 2. Materials and Methods

### 2.1. Study Design

We conducted a method comparison study with an observational design. Hospitalized patients admitted to the post-anesthesia care unit (PACU) of the University Medical Center Utrecht, the Netherlands, were continuously monitored during the first postoperative night following a surgical intervention. SpO_2_, PR, and RR were continuously monitored using both the earsensor and the routine bedside monitoring system. To ensure routine hospital care, treating clinicians did not have access to measurement data from the earsensor. Formal ethical approval for this study was obtained from the Medical Research Ethics Committee in Utrecht (MREC NL82135.000.23). Written informed consent was obtained prior to surgery.

### 2.2. Study Population and Setting

Hospitalized adult patients scheduled for an overnight stay at the PACU after intermediate and major surgery were eligible for inclusion. Exclusion criteria were planned or unplanned post-operative admission to the ICU, a surgical location or skin lesion close to the ear, pregnancy, and breastfeeding. Once admitted to the PACU, patients were routinely monitored (bedside routine standard), and the earsensor (index device) was applied by the researcher. Recording of the index vital signs with the earsensor was blinded.

### 2.3. Description of the Earsensor

**Figure 1:**
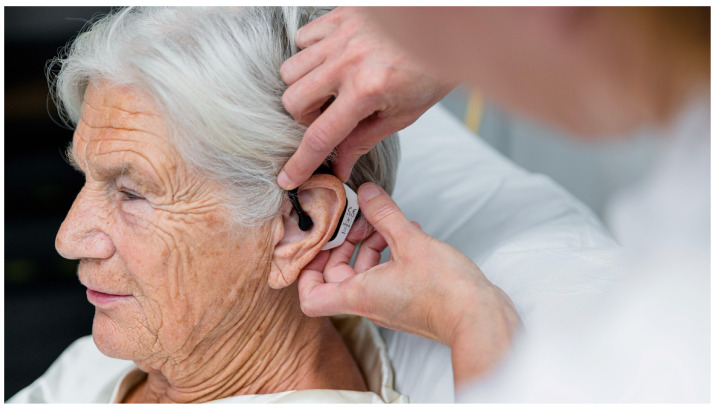
The earsensor (Vital Signs Monitoring System, FastFocus B.V., The Netherlands). The earsensor uses reflectance PPG to determine SpO_2_, PR, and RR. © 2024. Fastfocus B.V.

**Figure 2:**
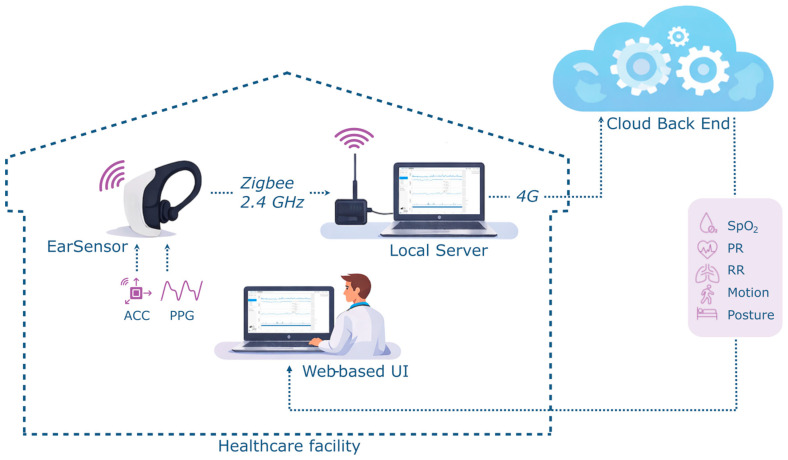
Schematic overview of the system architecture, illustrating the earsensor hardware components, data transmission pathways, and processing workflow.

The ear-worn sensor (Vital Signs Monitoring System, FastFocus B.V., Harmelen, The Netherlands) weighs 17 g and operates for at least 12 h after being fully charged (Figure 1). A three-axis accelerometer determines the patient’s posture and movement. The PR, RR, and SpO_2_ are derived using reflectance photoplethysmography (PPG) with a sampling frequency of 65 Hz. Both SpO_2_ and PR are measured using red and infrared LEDs every 2 min for 8 s, while RR (and PR) are measured using green and red LEDs every 5 min for a period of 20 s. Data quality is automatically assessed using an integrated quality index that excludes low-quality values caused by motion artifacts or poor signal-to-noise ratios. The earsensor transmits raw PPG to a receiver connected to a local hospital server. This server forwards the data to a secure cloud environment, where measurements are aggregated and processed by algorithms to compute all vital signs. Figure 2 provides a schematic overview of the system architecture.

### 2.4. Description of the Bedside Routine Standard

PR, RR, and SpO_2_ were continuously monitored using the index device (earsensor) and the reference device, a multiparameter bedside monitoring system designed for use in ICUs and operating rooms (XPREZZON, Spacelabs Healthcare, Snoqualmie, Washington, DC, USA). The reference device uses ECG for heart rate monitoring and thoracic impedance pneumography for RR. SpO_2_ was measured by pulse oximetry (NellCor OxiMax N-600X, Hayward, CA, USA). Both HR and RR were updated every 5 to 6 breaths or beats. Vital signs were averaged and stored approximately every minute (55 to 65 s).

### 2.5. Signal Analysis

Data from the earsensor and the reference system were retrieved in comma-separated (CSV) format and processed using MATLAB R2024b (The MathWorks, Natick, MA USA). Non-physiological outliers were removed: PR > 250 beats per minute (bpm), RR > 60 breaths per min (brpm), and SpO_2_ < 60%. Patient records with earsensor measurements of less than 1 h duration were excluded from analysis. Earsensor and reference data were synchronized to ensure temporal alignment. Data from the reference monitor were down-sampled and compared with the nearest time point of each value obtained from the earsensor to produce paired data points. No artifact removal was applied prior to the data analysis.

### 2.6. Outcomes and Statistical Analysis

The primary outcome was measurement bias and precision, expressed as 95% limits of agreement (LoA), for SpO_2_, PR, and RR. Accuracy was assessed using the average root mean square (ARMS) for each vital sign in accordance with ISO 80601-2-61:2017 [23] for pulse oximeters, although the earsensor is not solely a pulse oximetry system. RR and PR were considered clinically acceptable if within ≤3 breaths per minute (brpm) or ≤5 beats per minute (bpm), respectively [24,25]. For SpO_2_, measurements were considered acceptable if the bias was within ±2% of the reference standard and the ARMS ≤ 3% [23]. All paired data were analyzed using the Bland–Altman method for repeated measurements [26]. The LoA were calculated using a mixed effects model (MEM) with a modification to account for repeated measurements [27,28]. The MEM involves time as a random effect and adjusts for baseline, the average value of each patient over time, and the mean measurement between the earsensor and the reference system for each measurement.

Secondary outcomes included patient comfort and experience, assessed using a short questionnaire completed before the patient was transferred to the ward. The questionnaire (Table A1 in Appendix A) consisted of 7 questions scored on a 5-point Likert scale. Another secondary outcome was the technical performance of the earsensor, which was evaluated by the proportion of the total amount of data loss for each vital sign and maximum duration of data loss defined as gap durations with a maximum length of 5 min, 30 min, 60 min, 1–2 h, or longer than 2 h.

## 3. Results

From January to August 2024, a total of 28 patients were enrolled in the study, of whom 21 were included in the study. The other seven patients were excluded due to various reasons: technical failure of the system to connect (3 patients), two patients went directly to the ICU or ward instead of the PACU after surgery, one patient did not want to wear the earsensor after surgery, and one patient could not wear the earsensor due to agitation. Of the remaining 21 patients, one patient was excluded from the method comparison analyses since only half an hour of data was transmitted by the earsensor. In total, 264 h of vital signs monitoring on the PACU were available, with a median duration of 14 h per patient (minimum: 30 min; maximum: 17 h). Table 1 summarizes patient characteristics.

### 3.1. Oxygen Saturation

**Figure 3:**
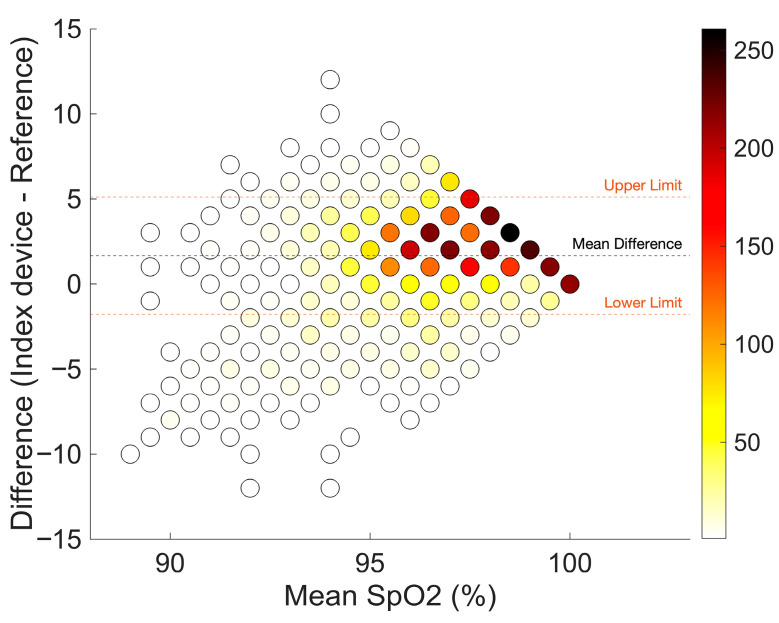
Bland–Altman plot of SpO_2_ measurements with few (white) to many (dark red) measurement pairs. The orange dashed line corresponds to the limits of agreement. Bias (mean difference) is shown as a black dashed line.

Table 2 shows the bias and precision (95% limits of agreement) of the comparison between the earsensor and the reference standard. A total of 4646 SpO_2_ measurement pairs were available for analysis. The earsensor overestimated oxygen saturation, with a bias (mean difference) of 1.7%, which was within the predefined acceptable range for clinical purposes. The LoA were considered wide (−1.8% to 5.1%), but the ARMS of 2.4% was within the predefined cutoff value, when considering a SpO_2_ range of 88–100%. Figure 3 illustrates the Bland–Altman plot with most readings obtained in the range of 95–100%. A very limited number of measurement pairs (n = 124; 2.7%) below <92% are shown in Figure 3.

### 3.2. Pulse Rate

**Figure 4:**
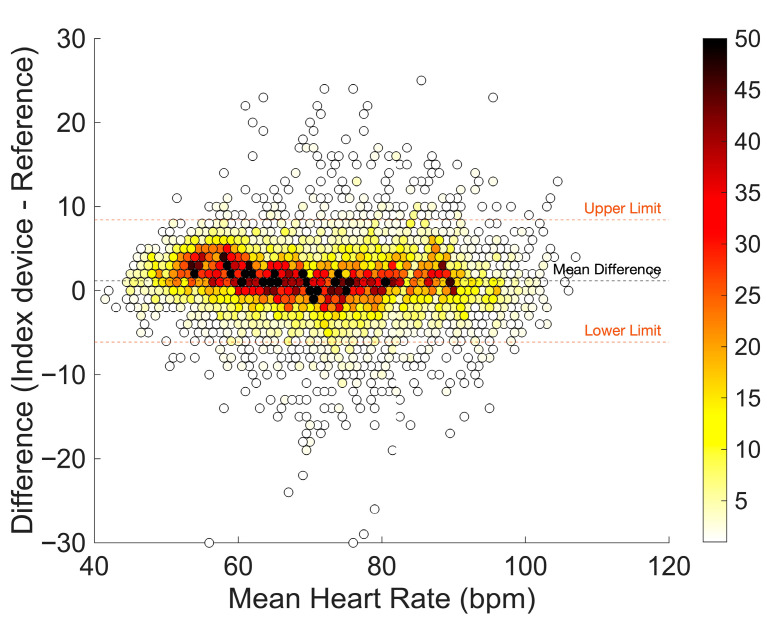
Bland–Altman plot of pulse rate measurements with few (white) to many (dark red) measurement pairs. The orange dashed line corresponds to the limits of agreement. Bias (mean difference) is shown as a black dashed line.

In total, 9354 measurement pairs were available for analysis. The bias was 1.2 bpm with LoA of −6.1 to 8.4 bpm, indicating sufficient accuracy and precision. The ARMS of 3.9 bpm was within the predefined cutoff value (Table 2). The mean difference varied between average pulse rate values. Overestimation of pulse rate was higher for average pulse rates below 60 bpm as opposed to higher pulse rate values (Figure 4).

### 3.3. Respiratory Rate

**Figure 5:**
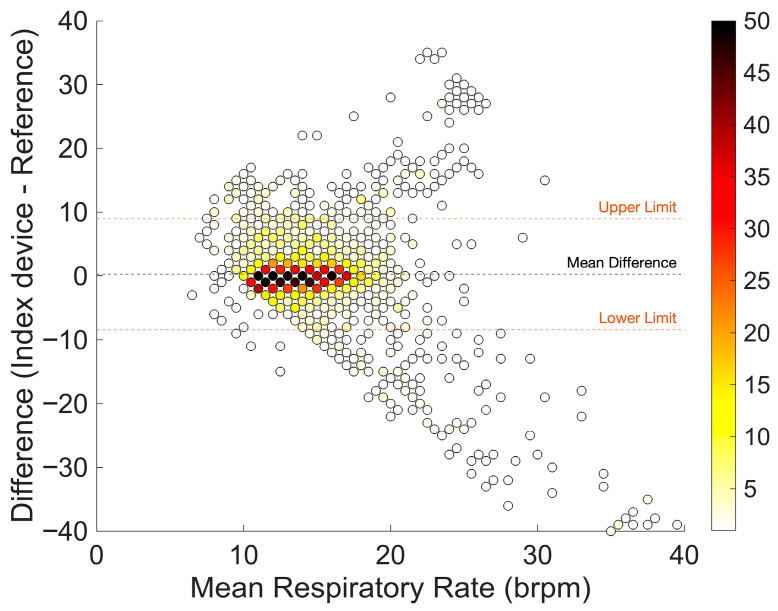
Bland–Altman plot of respiratory rate measurements with few (white) to many (dark red) measurement pairs. The orange dashed line corresponds to the limits of agreement. Bias (mean difference) is shown as a black dashed line.

A total of 2131 respiratory rate measurement pairs were available for analysis. The bias was low with 0.3 brpm. Both the LoA, with a range from −8.4 to 8.9, and ARMS of 3.9% were outside the predefined acceptable range for clinical purposes. Figure 5 indicates that the difference between the earsensor and the reference standard depends on the average breathing rate. Especially for respiratory rates above 20 breaths per minute, the values are outside the 95% LoA, and the values of the monitors differ considerably.

**Table 2:** Accuracy outcomes for all three vital signs.

	Number of Measurement Pairs	Bias	Lower LoA	Upper LoA	ARMS	SD
SpO_2_	4646	1.7	−1.8	5.1	2.4	1.8
Pulse rate	9354	1.2	−6.1	8.4	3.9	3.7
Respiratory rate	2131	0.3	−8.4	8.9	4.4	4.4

### 3.4. Technical Performance

**Figure 6:**
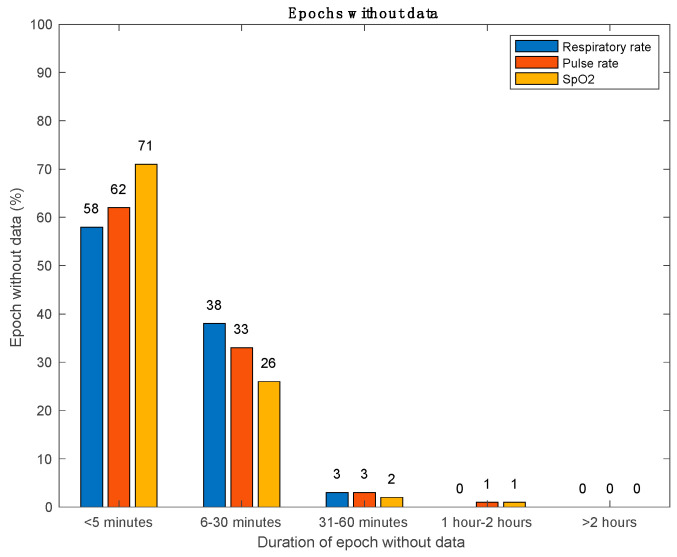
Overview of the number of epochs without data (%) divided over gaps of data loss with a maximum length of 5 min, 30 min, 1 h, 2 h, or longer than 2 h.

**Figure 7:**
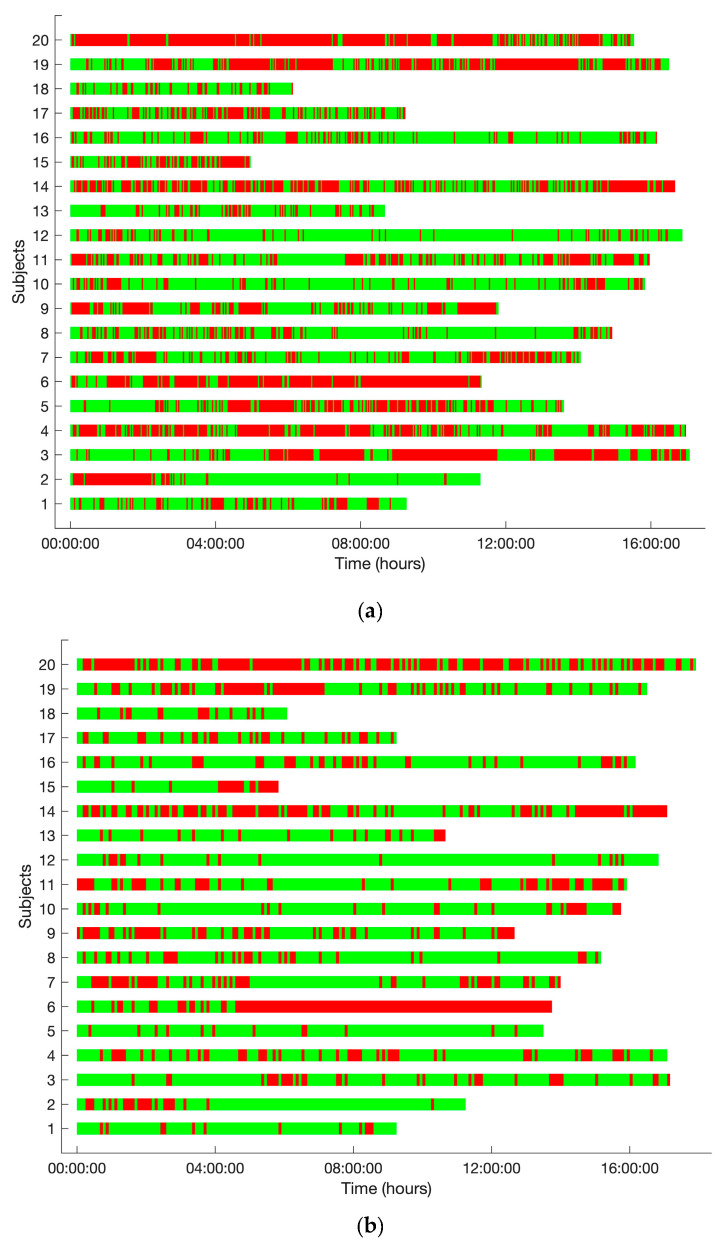

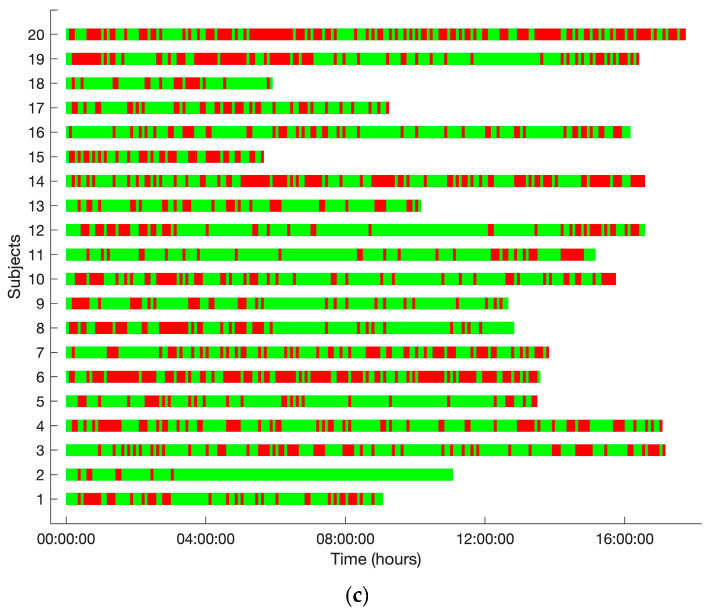
Data loss of all vital signs for each patient during the measurement period. Green represents available data. Red represents missing data. (**a**) Data loss of SpO_2_; (**b**) data loss of pulse rate; (**c**) data loss of respiratory rate.

Data loss of SpO_2_ measurements was 42%, whereas data loss for RR and PR were 33% and 29%, respectively. Data from the reference standard was not continuously available either, but with a lower missing data percentage of 0.3%, 2.2%, and 1.0% for SpO2, RR, and pulse rate, respectively. Figure 6 shows the percentage of the number of epochs without data divided over gaps of data loss with a maximum length of 5 min, 30 min, 60 min, 1 h, or longer than 2 h. Three percent or less of the gap durations were longer than 30 min for all three vital signs. The amount of data loss varied considerably among patients, as can be seen in Figure 7.

### 3.5. Patient Experiences

**Figure 8:**
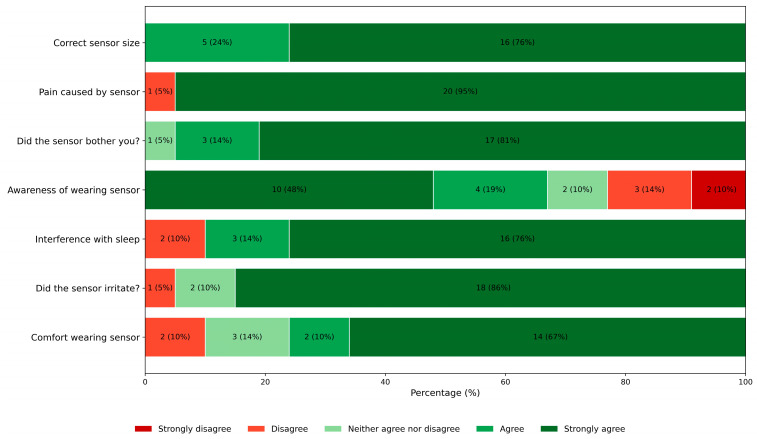
Overview of patient experiences.

Patient experiences were assessed using a short questionnaire (Figure 8). Overall, the comfort of wearing the sensor was rated high, with 77% of patients (16/21) reporting comfortable or very comfortable rates, but 2 patients felt the earsensor uncomfortable. The earsensor did not irritate the ear in the majority of patients (n = 18; 86%); one patient reported occasional irritation. For most patients (n = 19, 90%), the earsensor did not really interfere with sleep, whereas two patients (10%) reported sleep disturbance, especially when lying on the ear wearing the device. One patient mentioned that the sensor fell off during the night. More than half of the patients (n = 14; 67%) were not or not really aware of wearing the earsensor, but some patients (n = 5; 24%) were frequently or always aware while wearing it. In general, patients did not mind wearing the sensor (n = 20; 96%). Also, the sensor did not cause any pain in all patients (n = 20), except for one who reported some pain.

## 4. Discussion

This study evaluated the accuracy, technical performance, and patient comfort of a novel PPG-based earsensor for continuous monitoring of SpO_2_, PR, and RR in postoperative patients. The earsensor slightly overestimated SpO_2_, but measured SpO_2_ in accordance with ISO 80601-2-61 standards. Pulse rate was measured with high precision, whereas respiratory rate measurements showed a higher ARMS and fell outside the predefined acceptable range. Overall, patients reported high levels of comfort and acceptability even though they were recovering from surgery. Although most data gaps for each vital sign were shorter than 30 min, data availability varied substantially among patients overnight.

Three recent studies have evaluated the accuracy of SpO_2_ measurements using different PPG-based devices: a wristband (CardioWatch, Corsano Health, The Netherlands), an upper-arm device (ViQtor, SmartQare, The Netherlands), and a wearable finger probe (Radius PPG, Masimo, Irvine, CA, USA) in patients undergoing heart catheterizations or recovering from non-cardiac or trauma surgery in the PACU [18,19,29]. The wristband and finger probe demonstrated accurate SpO_2_ values with a bias of 0.54% (LoA: −3.1% to 4.0%) and 0.4% (LoA: −2.3% to 0.1%), respectively. These findings, however, cannot be directly compared with the present results, as those were conducted under more controlled conditions (e.g., immobile patients during catheterizations), included fewer measurement epochs (945 epochs of 28 s [19] compared to 4646 measurement pairs over 264 h within the present study), or covered only short monitoring durations [18].

Nonetheless, our findings are comparable to those of a recent validation of a PPG-based upper arm device (ViQtor, SmartQare, The Netherlands) conducted in the same PACU environment [29]. Compared with that device (bias: −0.03%; LoA: −4.14% to 4.09%), the earsensor in the present study showed a slight overestimation of SpO_2_, but with a narrower LoA [29]. Furthermore, our results align with those from a recent method comparison study in surgical patients in the ICU and high-dependency unit, which validated the CPC12S (Checkpoint Care, Kazanlak, Bulgaria) multiparameter system using a PPG earlobe sensor and the same reference system as in the present study [25].

The results of the present study confirm that the earsensor provides high accuracy for PR measurements, with a clinically acceptable LoA. Previous studies have reported substantial variation in the accuracy of PR measurements obtained using PPG-based sensors. For example, Monnink et al. [19] reported a smaller LoA for a PPG wristband during heart catheterizations, whereas van Melzen et al. [18] found a wider LoA using the Radius PPG, exceeding predefined cutoff values for PR. These differences may partly be explained by the diverse study settings, which ranged from immobile patients or bedridden patients to freely moving patients, where motion could compromise accuracy. It should also be noted that most wearable PPG sensors have not been tested in patients with atrial fibrillation or other arrhythmias [18]. Prior research described wearable PPG sensors as being accurate, except during episodes of atrial fibrillation, where they tend to underestimate the actual ventricular rate [30].

In contrast to SpO_2_ and PR, the accuracy of RR measured with the earsensor demonstrated wider LoA. This finding is consistent with other wearable device studies reporting large variability in RR measurements, suggesting that reliable RR estimation is more challenging than pulse rate monitoring [25,31]. However, it is important to recognize that the thoracic impedance measurements used as the reference cannot be considered the gold standard for RR monitoring, as they are prone to artifacts from patient movement and speech. Indeed, a direct comparison between capnography (the gold standard) and thoracic impedance in a similar PACU setting revealed poor agreement, with an ARMS of 5.39 brpm and a wide LoA (9.91–11.50 brpm) [29]. Therefore, part of the observed measurement error in RR of the present study likely reflects limitations of the reference standard rather than the earsenser itself.

It is known that cumbersome monitoring devices or wearable sensors, such as those incorporating finger pulse-oximeters or intermittent BP cuffs, can interfere with daily activity and sleep [32]. The results of our study show high patient comfort, which would facilitate adoption in clinical practice. However, it should be noted that these findings on high patient comfort were obtained over a relatively short period, during which patients may have remained drowsy due to the effects of surgery. A potential drawback of unobtrusive sensors is their current inability to measure a complete set of vital signs, which may necessitate additional manual measurements and limit integration into clinical practice [33]. However, ongoing advances in artificial intelligence and the findings from future implementation studies may help determine whether monitoring a limited subset of vital signs is sufficient for early detection of patient deterioration.

### Limitations

Several limitations of this study should be acknowledged. First, this study was conducted in a PACU environment, where the response time to physiological deterioration is considerably shorter than in general ward settings. Consequently, the number of measurement pairs with periods of hypoxemia <92% was limited. Validation of index devices under abnormal physiological ranges is challenging. Second, patients were monitored overnight following major surgery, which inherently limited patient movement. Despite this, substantial data loss occurred across all monitored vital signs. Another reason might be low peripheral perfusion or suboptimal sensor-skin contact between the sensor and skin that leads to a weak or irregular optical signal. As a result, the algorithm returns no values. It should be noted that the availability of vital signs measured with the earsensor and correct sensor placement was not routinely verified, precluding corrective adjustments that would typically occur in standard clinical practice when input is missing. Furthermore, the earsensor automatically excludes epochs with poor signal quality, often due to motion artifacts. Despite the expectation of limited patient movement during overnight postoperative recovery, substantial data loss (29–42%) was observed across all measured vital signs. In comparison, a recent validation study of a different PPG-based upper-arm sensor reported less than 10% missing data for each vital sign [29]. Nevertheless, the majority of data gaps (>97%) were shorter than 30 min, indicating that the earsensor was able to capture vital signs at a much higher frequency than routine manual observations. However, the potential impact of missing data on the usability of continuous vital sign monitoring remains unclear. Prolonged gaps in data availability (e.g., exceeding 30 min) may negatively affect clinicians’ trust in the system and could hinder its adoption in clinical practice. For future research, it would be valuable to define the minimum acceptable threshold for data availability, rather than focusing solely on missing data. Moreover, future studies should evaluate the ability of such sensors to reliably detect and track vital sign changes over time, rather than emphasizing beat-by-beat accuracy.

## 5. Conclusions

The tested wearable earsensor for continuous vital signs monitoring can accurately monitor SpO_2_ and pulse rate with performance meeting clinically acceptable limits, assessed during a relatively short period of recovery at night in postoperative patients. Respiratory rate measurements showed wider variability, with LoA and ARMS outside the predefined acceptable limits. Despite substantial data loss, patient-reported comfort and acceptability were high. Vital sign trend monitoring with PPG-based sensors could be a valuable tool for unobtrusive continuous monitoring in low-acuity settings. Future studies using the wearable earsensor are needed to evaluate the performance of continuous vital sign monitoring during prolonged use on the general ward.

## Figures and Tables

**Table 1:** Patient characteristics (N = 21).

Characteristic		Value
Gender, n (%)	Female	9 (43)
	Male	12 (57)
Age, years (median [IQR])		53 [40–66]
Type of surgery, n (%)	Neurosurgery	18 (86%)
	Vascular surgery	2 (9%)
	Urology	1 (5%)
Average duration of monitoring hours, median (range)		14 (0.5–17)
Total duration of monitoring, hours		264

## Data Availability

All data generated and analyzed during this study will be made available by the corresponding author on reasonable request (after anonymization).

## Appendix A

**Table A1:** Patient questionnaire.

How comfortable was it to wear the sensor? Very uncomfortable Very comfortable
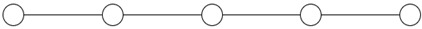
Did the sensor irritate your ear? Always irritating Not irritating
Did the sensor interfere with your sleep? Always Not at all
Were you aware of wearing the sensor? Not aware Always aware
Did you mind continuously wearing the sensor? Always Not at all
Did the sensor cause pain? Painful Not painful
Did the sensor have the correct size? Not at all Perfect fit

## References

[1] Schein R.M., Hazday N., Pena M., Ruben B.H., Sprung C.L. (1990). Clinical antecedents to in-hospital cardiopulmonary arrest. Chest.

[2] Jones D., Mitchell I., Hillman K., Story D. (2013). Defining clinical deterioration. Resuscitation.

[3] Goldhill D.R., McNarry A.F., Mandersloot G., McGinley A. (2005). A physiologically-based early warning score for ward patients: The association between score and outcome. Anaesthesia.

[4] Ludikhuize J., Smorenburg S.M., de Rooij S.E., de Jonge E. (2012). Identification of deteriorating patients on general wards; measurement of vital parameters and potential effectiveness of the Modified Early Warning Score. J. Crit. Care.

[5] Van Galen L.S., Struik P.W., Driesen B.E.J.M., Merten H., Ludikhuize J., Van Der Spoel J.I., Kramer M.H.H., Nanayakkara P.W.B. (2016). Delayed Recognition of Deterioration of Patients in General Wards Is Mostly Caused by Human Related Monitoring Failures: A Root Cause Analysis of Unplanned ICU Admissions. PLoS ONE.

[6] Eddahchouri Y., Koeneman M., Plokker M., Brouwer E., van de Belt T.H., van Goor H., Bredie S.J. (2021). Low compliance to a vital sign safety protocol on general hospital wards: A retrospective cohort study. Int. J. Nurs. Stud..

[7] Fuhrmann L., Lippert A., Perner A., Østergaard D. (2008). Incidence, staff awareness and mortality of patients at risk on general wards. Resuscitation.

[8] Beckett D., Gordon C., Paterson R., Chalkley S., Macleod D., Bell D. (2009). Assessment of clinical risk in the out of hours hospital prior to the introduction of Hospital at Night. Acute Med..

[9] Calzavacca P., Licari E., Tee A., Egi M., Downey A., Quach J., Haase-Fielitz A., Haase M., Bellomo R. (2010). The impact of Rapid Response System on delayed emergency team activation patient characteristics and outcomes—A follow-up study. Resuscitation.

[10] Kause J., Smith G., Prytherch D., Parr M., Flabouris A., Hillman K. (2004). A comparison of Antecedents to Cardiac Arrests, Deaths and EMergency Intensive care Admissions in Australia and New Zealand, and the United Kingdom—The ACADEMIA study. Resuscitation.

[11] Watkinson P.J., Pimentel M.A., Clifton L., Clifton D.A., Vollam S., Young D., Tarassenko L. (2020). Early detection of physiological deterioration in post-surgical patients using wearable technology combined with an integrated monitoring system: A pre-and post-interventional study. medRxiv.

[12] Downey C., Randell R., Brown J., Jayne D.G. (2018). Continuous Versus Intermittent Vital Signs Monitoring Using a Wearable, Wireless Patch in Patients Admitted to Surgical Wards: Pilot Cluster Randomized Controlled Trial. J. Med. Internet Res..

[13] Barker S.J., Shah N.K. (1997). The Effects of Motion on the Performance of Pulse Oximeters in Volunteers (Revised publication). Anesthesiology.

[14] Allen J. (2007). Photoplethysmography and its application in clinical physiological measurement. Physiol. Meas..

[15] Almarshad M.A., Islam M.S., Al-Ahmadi S., BaHammam A.S. (2022). Diagnostic Features and Potential Applications of PPG Signal in Healthcare: A Systematic Review. Healthcare.

[16] Saab R., Wu B.P., Rivas E., Chiu A., Lozovoskiy S., Ma C., Yang D., Turan A., Sessler D.I. (2021). Failure to detect ward hypoxaemia and hypotension: Contributions of insufficient assessment frequency and patient arousal during nursing assessments. Br. J. Anaesth..

[17] Charlton P.H., Bonnici T., Tarassenko L., Clifton D.A., Beale R., Watkinson P.J. (2016). An assessment of algorithms to estimate respiratory rate from the electrocardiogram and photoplethysmogram. Physiol. Meas..

[18] van Melzen R., Haveman M.E., Schuurmann R.C., van Amsterdam K., El Moumni M., Tabak M., Struys M.M.R.F., de Vries J.-P.P.M. (2024). Validity and Reliability of Wearable Sensors for Continuous Postoperative Vital Signs Monitoring in Patients Recovering from Trauma Surgery. Sensors.

[19] Monnink S.H.J., Vliet Mvan Kuiper M.J., Constandse J.C., Hoftijzer D., Muller M., Ronner E. (2024). Clinical evaluation of a smart wristband for monitoring oxygen saturation, pulse rate, and respiratory rate. J. Clin. Monit. Comput..

[20] Harskamp R.E., Bekker L., Himmelreich J.C.L., Clercq L.D., Karregat E.P.M., Sleeswijk M.E., Lucassen W.A.M. (2021). Performance of popular pulse oximeters compared with simultaneous arterial oxygen saturation or clinical-grade pulse oximetry: A cross-sectional validation study in intensive care patients. BMJ Open Respir. Res..

[21] Poorzargar K., Pham C., Ariaratnam J., Lee K., Parotto M., Englesakis M., Chung F., Nagappa M. (2022). Accuracy of pulse oximeters in measuring oxygen saturation in patients with poor peripheral perfusion: A systematic review. J. Clin. Monit. Comput..

[22] Saugel B., Hoppe P., Khanna A.K. (2020). Automated Continuous Noninvasive Ward Monitoring: Validation of Measurement Systems Is the Real Challenge. Anesthesiology.

[23] (2017). Medical Electrical Equipment—Part 2–61: Particular Requirements for Basic Safety and Essential Performance of Pulse Oximeter Equipment.

[24] Haahr-Raunkjaer C., Skovbye M., Rasmussen S.M., Elvekjaer M., Sørensen H.B.D., Meyhoff C.S., Aasvang E.K. (2022). Agreement between standard and continuous wireless vital sign measurements after major abdominal surgery: A clinical comparison study. Physiol. Meas..

[25] Breteler M.J.M., Leigard E., Hartung L.C., Welch J.R., Brealey D.A., Fritsch S.J., Konrad D., Hertzberg D., Bell M., Rienstra H. (2025). Reliability of an all-in-one wearable sensor for continuous vital signs monitoring in high-risk patients: The NIGHTINGALE clinical validation study. J. Clin. Monit. Comput..

[26] Bland J.M., Altman D.G. (2007). Agreement between methods of measurement with multiple observations per individual. J. Biopharm. Stat..

[27] Myles P.S., Cui J. (2007). Using the Bland-Altman method to measure agreement with repeated measures. Br. J. Anaesth..

[28] Parker R.A., Weir C.J., Rubio N., Rabinovich R., Pinnock H., Hanley J., McCloughan L., Drost E.M., Mantoani L.C., MacNee W. (2016). Application of Mixed Effects Limits of Agreement in the Presence of Multiple Sources of Variability: Exemplar from the Comparison of Several Devices to Measure Respiratory Rate in COPD Patients. PLoS ONE.

[29] Reijmers N., van Kootwijk A., de Waal E.E.C. (2025). Accuracy of vital sign monitoring using a photoplethysmography upper arm wearable device in postoperative non-cardiac surgery patients: A prospective observational clinical validation study. J. Clin. Monit. Comput..

[30] Breteler M.J., KleinJan E.J., Dohmen D.A., Leenen L.P., van Hillegersberg R., Ruurda J.P., van Loon K., Blokhuis T.J., Kalkman C.J. (2020). Vital Signs Monitoring with Wearable Sensors in High-risk Surgical Patients: A Clinical Validation Study. Anesthesiology.

[31] van der Stam J.A., Mestrom E.H., Scheerhoorn J., Jacobs F.E., Nienhuijs S., Boer A.K., Riel N.A.W.V., de Morree H.M., Bonomi A.G., Scharnhorst V. (2023). The Accuracy of Wrist-Worn Photoplethysmogram-Measured Heart and Respiratory Rates in Abdominal Surgery Patients: Observational Prospective Clinical Validation Study. JMIR Perioper. Med..

[32] Leenen J.P.L., Leerentveld C., van Dijk J.D., van Westreenen H.L., Schoonhoven L., Patijn G.A. (2020). Current Evidence for Continuous Vital Signs Monitoring by Wearable Wireless Devices in Hospitalized Adults: Systematic Review. J. Med. Internet Res..

[33] Leenen J.P.L., Rasing H.J.M., Kalkman C.J., Schoonhoven L., Patijn G.A. (2023). Process Evaluation of a Wireless Wearable Continuous Vital Signs Monitoring Intervention in 2 General Hospital Wards: Mixed Methods Study. JMIR Nurs..

